# Regulation of Mitoflash Biogenesis and Signaling by Mitochondrial Dynamics

**DOI:** 10.1038/srep32933

**Published:** 2016-09-13

**Authors:** Wenwen Li, Tao Sun, Beibei Liu, Di Wu, Wenfeng Qi, Xianhua Wang, Qi Ma, Heping Cheng

**Affiliations:** 1 State Key Laboratory of Membrane Biology, Beijing Key Laboratory of Cardiometabolic Molecular Medicine, Peking-Tsinghua Center for Life Sciences, Institute of Molecular Medicine, Peking University, Beijing, China

## Abstract

Mitochondria are highly dynamic organelles undergoing constant network reorganization and exhibiting stochastic signaling events in the form of mitochondrial flashes (mitoflashes). Here we investigate whether and how mitochondrial network dynamics regulate mitoflash biogenesis and signaling. We found that mitoflash frequency was largely invariant when network fragmentized or redistributed in the absence of mitofusin (Mfn) 1, Mfn2, or Kif5b. However, Opa1 deficiency decreased spontaneous mitoflash frequency due to superimposing changes in respiratory function, whereas mitoflash response to non-metabolic stimulation was unchanged despite network fragmentation. In Drp1- or Mff-deficient cells whose mitochondria hyperfused into a single whole-cell reticulum, the frequency of mitoflashes of regular amplitude and duration was again unaltered, although brief and low-amplitude “miniflashes” emerged because of improved detection ability. As the network reorganized, however, the signal mass of mitoflash signaling was dynamically regulated in accordance with the degree of network connectivity. These findings demonstrate a novel functional role of mitochondrial network dynamics and uncover a magnitude- rather than frequency-modulatory mechanism in the regulation of mitoflash signaling. In addition, our data support a stochastic trigger model for the ignition of mitoflashes.

Mitochondria are highly dynamic eukaryotic organelles which display continuous fusion, fission, and movement such that intracellular network of mitochondria reorganizes and redistributes constantly^1^,^2^. Such morphological dynamics of mitochondria are thought to be critical for the maintenance of mitochondrial functions in energy production and metabolism and in the regulation of multiple biological progresses including mitochondrial genomic stability, calcium signaling, redox balance, apoptosis, and mitochondrial quality control^3^,^4^,^5^. Dysregulation of mitochondrial dynamics has been linked to neurodegenerative diseases, cancers and ageing^1^,^6^,^7^,^8^.

In mammalian cells, mitochondrial dynamics are driven and regulated by a number of GTPases, including mitofusins (Mfn), Opa1, Drp1 and Mff, and a variety of motor proteins such as kinesins. Outer mitochondrial membrane (OMM)-bound GTPases Mfn1 and Mfn2 and the dynamin-related GTPase Opa1 in the inner mitochondrial membrane (IMM) coordinately regulate the fusion of OMM and IMM, respectively, in the double-membrane organelle^6^,^9^. Mitochondrial fission requires another dynamin-related GTPase Drp1 that locates primarily in the cytoplasm and is recruited to the surface of OMM by binding to the protein Mff, where it self-assembles into spirals that wraps around mitochondria and, as a mechanochemical enzyme, pinches off their membranes^11^,^12^. Kinesins play key roles in moving organelles within cells. A member of their family, Kif5b, is essential for mitochondrial motility by transporting the organelle along microtubules^14^,^15^.

Recently, we and others have shown that respiring mitochondria exhibit sudden and transient chemical and electrical excitation, a phenomenon originally called “superoxide flash” and now known as “mitochondrial flash” or “mitoflash”^16^,^17^,^18^,^19^. A mitoflash comprises diverse signals including a burst of superoxide production, a transient alkalization of the matrix, a reversible oxidation of NADH and FADH_2_, and an action potential-like electrical excitation of IMM, all occurring concurrently over a 10-s time scale^16^,^17^,^20^,^21^. Emerging evidence indicates that mitoflashes constitute elemental mitochondrial signaling events that participate in vital physiological processes ranging from metabolism to stress responses to cell fate regulation^22^,^23^,^24^,^25^,^26^. The occurrence of mitoflashes depends on mitochondrial respiratory function and is likely controlled by transient opening of the mitochondrial permeability transition pore (mPTP). Consistent with its signaling role, mitoflash biogenesis is highly regulated in a frequency-modulatory (FM) manner, with activators including mitochondrial Ca^2+^, oxidants such as reactive oxygen species (ROS), and hyperosmotic stress; and depressors including ROS scavengers and mPTP inhibitors^16^,^24^,^27^,^28^. However, an important yet unanswered question is whether and how network dynamics interact with the newly-discovered functional dynamism, mitoflashes, which represents fundamental mitochondrial signaling events.

Here we investigate possible roles of mitochondrial dynamics in the regulation of mitoflash biogenesis and signaling by manipulating key dynamic proteins that mediate mitochondrial fission, fusion, and motility in a variety of cells. We performed parallel respirometric measurement and examined mitoflash responses to non-metabolic stimulations to appraise and separate contributions from altered respiratory function, if any. Our results indicate that, when mitochondrial network reorganizes or redistributes, mitoflash frequency remains constant at the cellular level provided that respiratory function is intact; at the same time, mitoflash signal mass is enhanced or decreased when mitochondrial network hyperfuses or fragmentizes, respectively. These data are unified in a stochastic trigger model of mitoflash biogenesis and uncover that changing mitochondrial network affords a magnitude-modulatory rather than FM mechanism in the regulation of mitoflash signaling.

## Results

### Mitofusin deficiency does not alter mitoflash frequency

**Figure 1:**
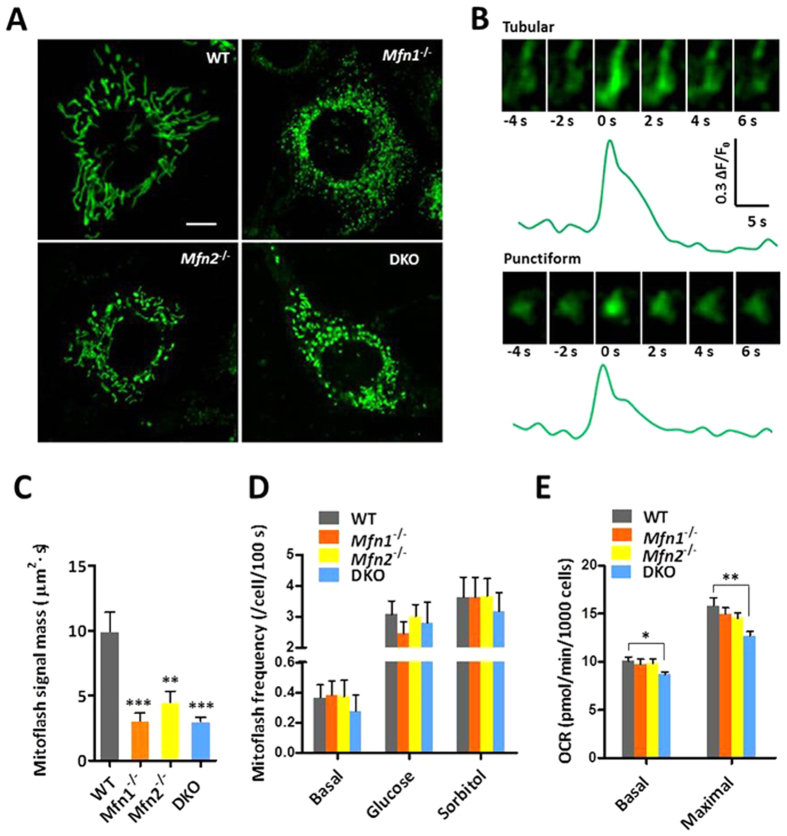
Mitoflash activity in mitofusin-deficient MEFs with fragmented mitochondrial network. **(A)** Mitochondrial morphology in wild-type (WT), Mfn1-knockout, Mfn2-knockout and Mfn1/2 double-knockout (DKO) MEFs. Scale bar, 5 μm. **(B)** Time-lapse views and time course plot of representative mitoflashes in WT (top) and Mfn1/2 DKO MEFs (bottom). Horizontal size of images, 2.5 μm. **(C)** Mitoflash signal masses in different groups. n = 35–36 mitoflashes per group. **p < 0.01; ***p < 0.001 vs WT cells. **(D)** Spontaneous and hyperosmosis (270 mM glucose or sorbitol)-stimulated mitoflash activities in the absence of either or both mitofusins. n = 22–52 cells per group. **(E)** Mitochondrial metabolic efficiency in mitofusin-knockout cells. OCR, oxygen consumption rate. n = 3 independent experiments. *p < 0.05; **p < 0.01.

We first examined mitoflash activity in response to genetic manipulations of Mfn1 and Mfn2, both are crucial for promoting the OMM fusion. In mouse embryonic fibroblasts (MEFs) of wild-type, *Mfn1*^−/−^, *Mfn2*^−/−^, and Mfn1/2 double-knockout (DKO) background, mitochondrial targeted, circularly permuted yellow fluorescent protein (mt-cpYFP) was expressed as the mitoflash biosensor, the fluorescence of which increases upon bursting superoxide production and transient alkalization^16^,^29^. As expected, deficiency of either or both mitofusins transformed mitochondrial morphology from the mostly tubular and reticular network in wild-type cells to a severely fragmented network consisting exclusively of punctiform mitochondria of round or oval appearance (Fig. 1A). Time-lapse imaging of wild-type MEFs revealed spontaneous and quantal mitoflashes, and individual events exhibited punctiform, tubular or reticular appearance, with the tubular ones being predominant (Fig. 1B). Mitoflash activity persisted in all three types of Mfn-deficient cells, but they were all confined to single punctiform mitochondria. The rate of mitoflash occurrence was similar in wild-type and Mfn-deficient cells, at a level of about 0.4 events/cell/100 s (Fig. 1D). Parametric measurement showed that their spatial area and signal mass (amplitude × duration × area) were decreased by 55–70% in the absence of either or both mitofusins, while their amplitude and duration were unchanged (Fig. 1C and Supplementary information, Figure S1). As mitoflash area is related to mitochondrial functional connectivity^23^, the composite parameter “signal mass” is preferred in the context of mitoflash signaling. When cells were challenged with hyperosmotic stress (270 mM glucose or sorbitol) to elevate its mitoflash activity via a non-metabolic mechanism^28^, nearly identical mitoflash frequency was attained, reaching ~3.0 events/cell/100 s, regardless of mitofusin deficiency. By measuring the oxygen consumption rate (OCR) using the Seahorse XF24 analyzer, we found that basal metabolism and maximal oxidative capacity remained unchanged with deficiency of either mitofusin, but exhibited mild decreases (14–20%) in Mfn1/2 DKO cells (Fig. 1E), the latter in agreement with previous findings^30^. These results indicate that the rate of occurrence of spontaneous or stimulated mitoflashes at the cellular level is largely independent of OMM fusion in the present experimental conditions.

### Mitochondrial motility is dispensable for mitoflash biogenesis

**Figure 2:**
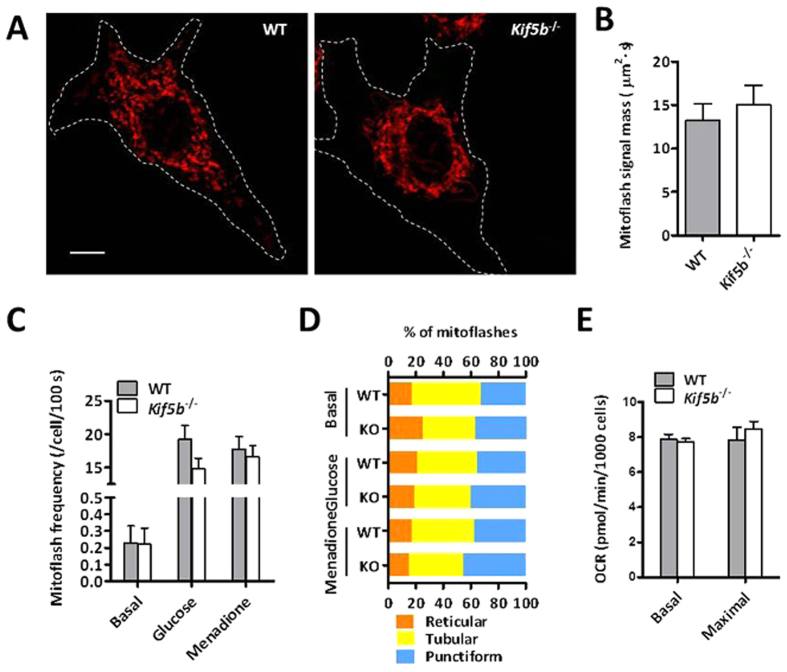
Effects of Kif5b on mitochondrial distribution and mitoflash activity. **(A)** Subcellular distribution of mitochondria in wild-type (WT) and Kif5b-knockout (KO) NRK cells. White dashed lines track cell boundaries seen in DIC images. Scale bar, 5 μm. **(B)** Mitoflash signal masses in WT and *Kif5b*^−/−^ cells. **(C)** Spontaneous and evoked mitoflash frequencies in the presence and absence of Kif5b. n = 13–26 cells/group. **(D)** Mitoflash morphology in WT and *Kif5b*^−/−^ cells under different conditions. Glucose, 270 mM; menadione, 80 μM. n = 42–51 mitoflashes per group. **(E)** Mitochondrial metabolic efficiency. n = 3 independent experiments.

Mitochondrial movement along microtubules, which is predominantly mediated by Kif5b, is essential for mitochondrial fusion and intracellular mitochondrial distribution^14^. Next, we examined mitoflash activity in normal rat kidney (NRK) cells in which mitochondrial motility and consequentially mitochondrial fusion are completely abolished with Kif5b knockout^15^. Mitoflashes displayed a range of morphological shapes in wild-type NRK cells, punctiform, tubular, or reticular (Supplementary information, Figure S2A), with their average spatial areas of 0.68, 2.41 and 4.10 μm^2^ and their relative percentages of 33, 44 and 23%, respectively (Fig. 2D and Supplementary information, Figure S2B). In *Kif5b*^−/−^ cells with densely packed perinuclear mitochondrial network (Fig. 2A), the same three types of mitoflashes occurred with their respective proportions and spatial areas all similar as those in wild-type cells (Fig. 2D and Supplementary information, Figure S2B), and their combined mitoflash signal mass and frequency was also unchanged (Fig. 2B,C). Furthermore, loss of Kif5b did not alter mitoflash response to either hyperosmotic stimulation by 270 mM glucose or oxidative stimulation by 80 μM menadione (Fig. 2C). The distribution of stress-induced mitoflashes among three morphological categories was not changed, either (Fig. 2D). Parallel experiments revealed that respiratory function remained intact in *Kif5b*-null cells (Fig. 2E). Thus, mitochondrial motility as well as mitochondrial fusion does not play any major role in the regulation of mitoflash biogenesis and its stress responses.

### Opa1 deficiency suppresses mitoflashes and mitochondrial respiration

**Figure 3:**
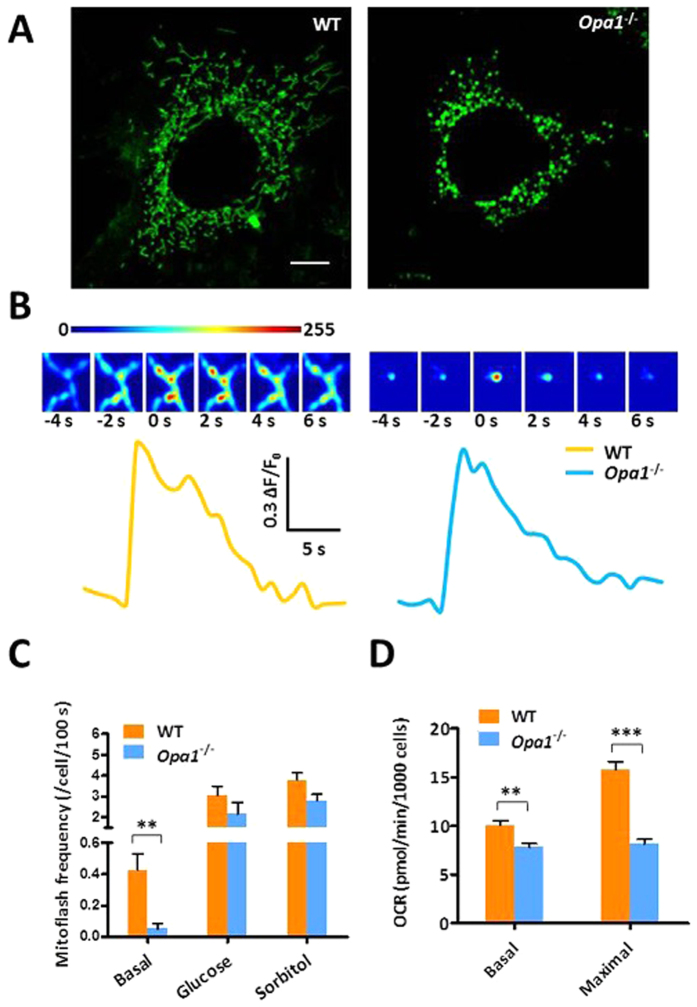
Mitoflash activity in fragmented mitochondria network in Opa1-knockout MEFs. **(A)** Mitochondrial morphology in wild-type (WT) and Opa1-knockout (KO) cells expressing mt-cpYFP. Scale bar, 5 μm. **(B)** Time courses of mitoflashes in WT and Opa1-KO cells. Insets show enlarged time-lapse images of the mitoflashes with horizontal size of 3.6 μm. **(C)** Effect of Opa1 knockout on spontaneous and stimulated mitoflash activity. Note that while spontaneous basal activity was suppressed, mitoflash response to hyperosmotic stimulation remained intact in the absence of Opa1. n = 28–40 cells per group. **(D)** Mitochondrial metabolic efficiency. n = 3 independent experiments. **p < 0.01; ***p < 0.001.

Recently, Santo-Domingo *et al*. have shown that knockout of Opa1, an IMM fusion protein, completely abolished mitoflashes (named “pH flashes” that were indicated by the probe mitoSypHer in their report) in MEFs; and, based on this finding, they proposed a model in which mitoflash biogenesis is causatively linked to the events of IMM fusion^31^. We therefore revisited Opa1 effect on mitoflash activity in MEFs. As expected, *Opa1*^−/−^ MEFs displayed aberrant mitochondrial morphology: they were all dispersed as punctiform mitochondria (Fig. 3A). Nonetheless, we still recorded discrete punctiform mitoflashes occurring in these cells, albeit at a markedly decreased frequency. The mitoflash frequencies in the presence and absence of Opa1 were 0.43 and 0.05 events/cell/100 s, respectively (Fig. 3C). Individual mitoflashes in wild-type and *Opa1*^−/−^ cells were indistinguishable in terms of average amplitude and kinetics (Fig. 3B and Supplementary information, Figure S3), indicating that the machinery of mitoflash biogenesis is still functional in the absence of Opa1. Using the ratiometric pH indicator mitoSypHer as reported in the work of Santo-Domingo *et al*.^31^, we consistently observed spontaneous mitoflashes in Opa1-deficient cells (Supplementary information, Figure S4A,B). Moreover, hyperosmotic stress also elevated the mitoSypHer-reported mitoflash activity (Supplementary information, Figure S4C).

The very presence of mitoflashes of normal amplitude and time course in *Opa1*^−/−^ MEFs strongly suggests that Opa1 regulates mitoflash biogenesis via a mechanism other than regulating IMM fusion. In this regard, it has been shown that Opa1 plays a key role in regulating mitochondrial respiratory function because it alters the IMM crista structure, the respiratory supercomplex assembly, and hence the metabolic efficiency^32^,^33^. Direct respirometric measurement confirmed that loss of Opa1 significantly attenuated mitochondrial basal respiration and maximal oxidative capacity by 22.8 and 48.7%, respectively, in MEFs (Fig. 3D). This result lends support to the hypothesis that Opa1 deficiency secondarily impairs the biogenesis of spontaneous mitoflashes via a direct suppression of respiratory activity. To further test this possibility, we treated the wild-type cells with the respiratory chain inhibitors, including NaN3, oligomycin, and rotenone. These metabolic inhibitors attenuated the mitoflash frequency by ~90% while depressing the metabolic rate by ~50% (Supplementary information, Figure S5A,B). Concomitantly, mitochondrial membrane potential was similarly depolarized by ~50% in Opa1-mutant cells and in the presence of these metabolic inhibitors (Supplementary information, Figure S5C,D). These results indicate that inhibition of spontaneous mitoflash activity in *Opa1*-null cells can be largely accounted for by the depressed metabolic rate and membrane potential. Furthermore, if this is the case, mitoflash response to stimuli bypassing metabolism might remain intact even in the absence of Opa1. Strikingly, we found that mitoflash frequency was greatly elevated by hyperosmotic stimulations in Opa1-deficient cells, to a level similar to that in wild-type cells under the same conditions (Fig. 3C). In other words, the lack of the pro-fusion protein Opa1 does not compromise the capacity of mitoflash response to hyperosmotic stress, excluding IMM fusion as a prerequisite of mitoflash biogenesis.

### Spatial synchrony in hyperfused mitochondria

**Figure 4:**
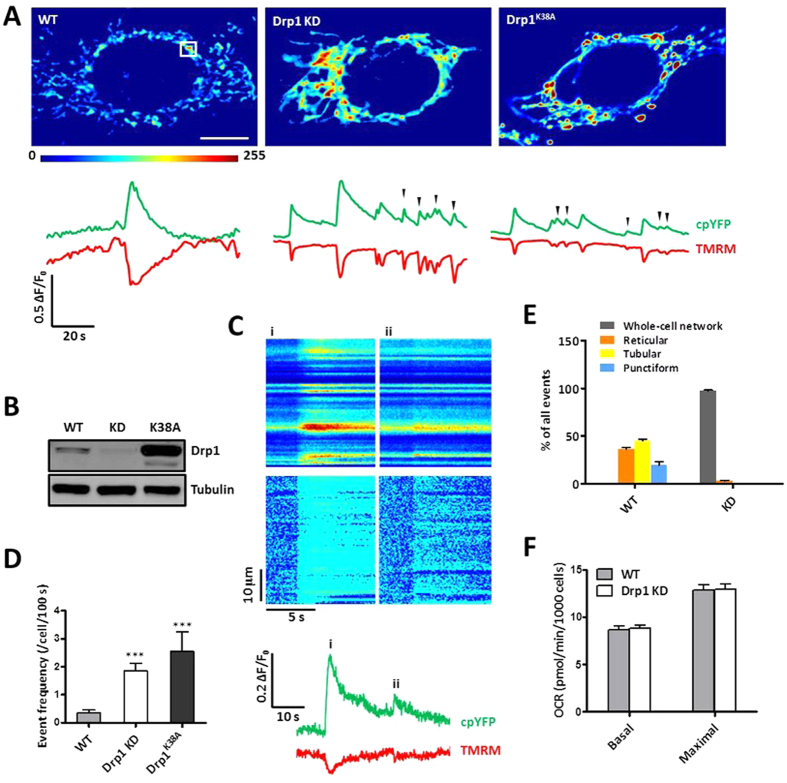
Mitoflash activity in hyperfused mitochondria. **(A)** Representative shapes and time courses of mitoflashes in wild-type (WT), Drp1-knockdown (KD) and Drp1^K38A^-expressing HeLa cells. Upper panels: Confocal images of mitoflashes in single mitochondrion (left, marked by box) and whole-cell mitochondrial reticulum (middle and right) at their peak intensities. Scale bar, 5 μm. Bottom panels: corresponding time course plots of simultaneously recorded mt-cpYFP and TMRM fluorescent signals. Note frequent miniflashes of small amplitudes and short durations (marked by arrows) in Drp1-KD and Drp1^K38A^ HeLa cells. See also Supplementary information, Movie S1. **(B)** Western blot analysis of Drp1 knockdown and Drp1^K38A^ expression. **(C)** Spatial synchrony of mitoflashes in whole-cell mitochondrial reticulum in a Drp1-KD cell. Upper panels: Kymographs of raw and normalized (F/F_0_) mt-cpYFP fluorescence along a line placed across the cell, showing synchronous ignition and evolvement of whole-cell mitochondrial reticulum in two consecutive events of different amplitudes (events i and ii). Bottom panel: time courses of mitoflashes reported by mt-cpYFP and TMRM simultaneously. Linescan imaging was performed at 5 ms/line. **(D)** Event frequency in WT, Drp1-KD and Drp1^K38A^ cells. n = 23–33 cells in each group. ***p < 0.001 vs WT cells. **(E)** Morphology of mitoflashes in control and Drp1-KD cells. n = 33–91 mitoflashes per group. **(F)** Mitochondrial metabolic efficiency in control and Drp1-KD cells. n = 3 independent experiments.

Next, we sought to address whether and how mitochondrial hyperfusion regulates mitoflash frequency and signal mass. For this purpose, we knocked-down the pro-fission protein Drp1 using RNA interference (RNAi) or transiently expressed its dominant negative Drp1^K38A^ mutant^34^ in HeLa cells stably expressing mt-cpYFP. Both Drp1 depletion and Drp1^K38A^ expression^31^ promoted hyperfusion of mitochondria into extended tubular networks (Fig. 4A,B). Remarkably, mitoflash imaging revealed that, in >78% cells, the entire intracellular mitochondrial network underwent synchronous activities (Fig. 4A and Supplementary information, Movie S1), with average area of 166.4 ± 12.0 μm^2^ (n = 124 cells), indicating that all mitochondria were fused into a single functional unit, similar to large mitochondrial reticulum revealed by mitoflash imaging in mouse skeletal muscles *in vivo*^23^ and *in vitro*^18^.

To determine spatial synchrony of whole-cell mitoflash activation, we performed linescan at 200 Hz and two consecutive events of different amplitudes are shown in Fig. 4C. It is clear that both events were activated abruptly and synchronously across the entire dimension of the cell. Even by linescan imaging at 763 μs/line (Supplementary information, Figure S6), we failed to resolve any local delays in the rising phase of mitoflashes over a distance of tens of micrometers. This result argues against the possibility of diffusible chemicals as the primary synchronizer, and provides compelling evidence that synchronization within an extended mitochondrial reticulum is controlled by the spreading of electrophysiological signals (e.g., membrane depolarization)^23^,^35^. Using the cable theory^36^, we estimated that the electrical length constant is ~82 μm for a contrived tubular mitochondrion (Supplementary information, Table S1), a value that is consistent with an instantaneous and near uniform distribution of electrical signals over the whole-cell reticulum.

### Mitoflashes and miniflashes in mitochondria-hyperfused cells

**Figure 5:**
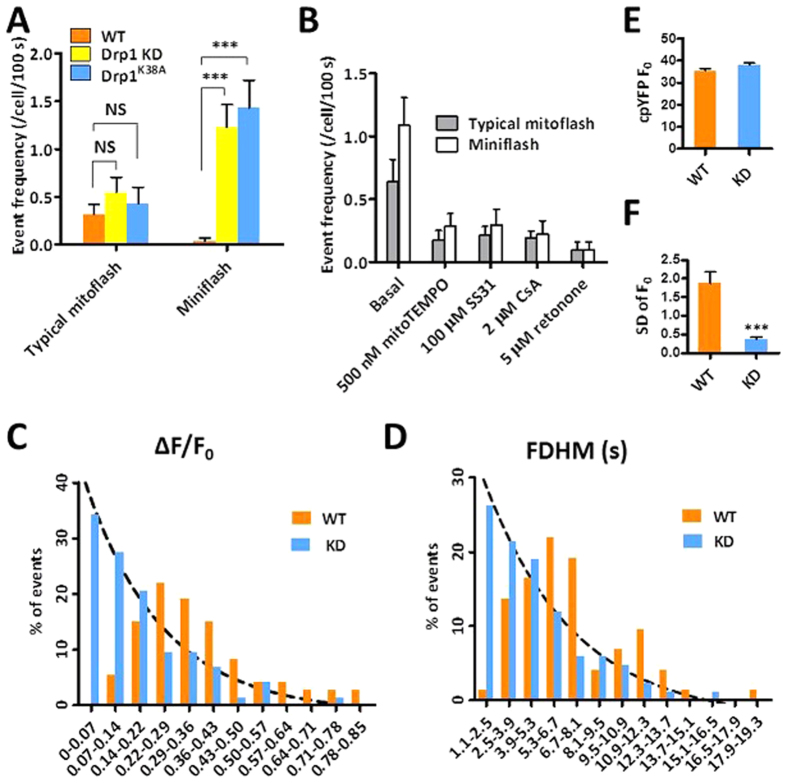
Properties of individual mitoflash events in mitochondria-hyperfused cells. (**A**) Event frequency in wild-type (WT), Drp1-knockdown (KD) and Drp1^K38A^-expressing HeLa cells. n = 22–30 mitoflashes in each group. **(B)** Mitoflash activity in Drp1-KD cells was inhibited by ROS scavengers (mitoTEMPO and ss31), mPTP inhibitor (CsA) and respiratory chain inhibitor (rotenone). n = 21–52 cells/group. **(C**,**D)** Histograms of event amplitude (ΔF/F_0_) (**C**) and duration (full duration at half maximum, FDHM) (**D**). Dashed line represents a single-exponential fit to the data from Drp1-KD cells. **(E)** Basal cpYFP fluorescence intensity. n = 64–77 cells/group. **(F)** Improved signal-to-noise ratio for event detection in Drp1-KD versus control group. n = 24–29 events/group. NS, no significance; ***p < 0.001.

Visual examination and quantitative measurement of whole-cell mitoflashes revealed another striking finding - the appearance of a subpopulation of events of tiny amplitude and brief duration, or “miniflashes”, in hyperfused mitochondrial reticulum (Fig. 4A). For the purpose of bisecting all events into two categories, we classified miniflashes as those with ΔF/F_0_ < 0.15 and FDHM (full-duration at half maximum) <4 s. Miniflashes, which were rare in control cells dominated over mitoflashes in both Drp1-knockdown (KD) and Drp1^K38A^-expressing cells (Fig. 5A). When mitoflashes and miniflashes were combined, the frequency of all events was markedly elevated in these mitochondria-hyperfused cells (Fig. 4D). However, when comparison was confined to the mitoflash subgroup, we found that regular mitoflash frequency was unaffected in cells with hyperfused mitochondria (Fig. 5A). Notably, robust miniflash activity was also present when mitochondrial hyperfusion was induced with a small molecule S-3 that enhances mitofusin activity^37^ or by depletion of Mff (Supplementary information, Figure S7), another essential factor for mitochondrial fission^10^.

At the first glance, the above results suggest that network hyperfusion promotes miniflash production without altering regular mitoflash activity as well as respiration (Fig. 4F). However, a caveat is that, before we reach that conclusion, we should determine whether mitoflashes and miniflashes are of the same or different origin. In this regard, we showed that both mitoflashes and miniflashes were accompanied by transient depolarization of the mitochondrial membrane potential (ΔΨ_m_) (Fig. 4A). They were equally sensitive to inhibition by ROS scavengers (e.g., mitoTEMPO and SS31), the mPTP inhibitor (CsA), and the respiratory chain inhibitor (rotenone) (Fig. 5B). Moreover, we found that histograms for the amplitude and duration of all events were well-fitted to single-exponential functions, with decay constants of 0.22 (ΔF/F_0_) and 5.57 s (FDHM), respectively (Fig. 5C,D). Together with the shared feature of spatially synchronous activation, mitoflashes and miniflashes appear to belong to a single population of events, the parameters of which displaying continuous distributions.

If miniflashes and mitoflashes are mechanistically identical, then the emergence of miniflashes in Drp1-inhibited cells could be only apparent, an epiphenomenal associated with hyperfusion. To this end, we noticed that, basal mt-cpYFP signals (F_0_), spatially averaged over the entire mitoflash area, displayed similar absolute values in both control and Drp1-inhibited groups, but an 81% smaller standard deviation (SD), equivalent to a 5.2-fold higher signal-to-noise ratio (SNR) in cells harboring whole-cell mitochondrial reticulum (Fig. 5E,F). As such, miniflashes would mostly go undetected in control group but would be readily resolved in Drp1-inhibtied group; nevertheless, mitoflashes of regular size and duration would be faithfully detected in both groups. Since regular mitoflashes should display similar detectability and their frequency was unchanged in wild-type versus Drp1-defective cells, we interpret that mitochondrial hyperfusion does not alter the rate of occurrence of mitoflashes in a cell, and the apparent increase in all-event frequency is attributable to greatly improved detectability for small and brief events. It is noteworthy that the mitoflash signal mass and the frequency per mitochondrion were both dramatically increased when all mitochondria fused into one single reticulum in a cell. Furthermore, we found that the Drp1-KD and control cells exhibited similar levels of mitochondrial respiratory function (Fig. 4F), in general agreement with previous reports^38^.

### Local swelling at preferred sites in mitoflashes of hyperfused mitochondria

**Figure 6:**
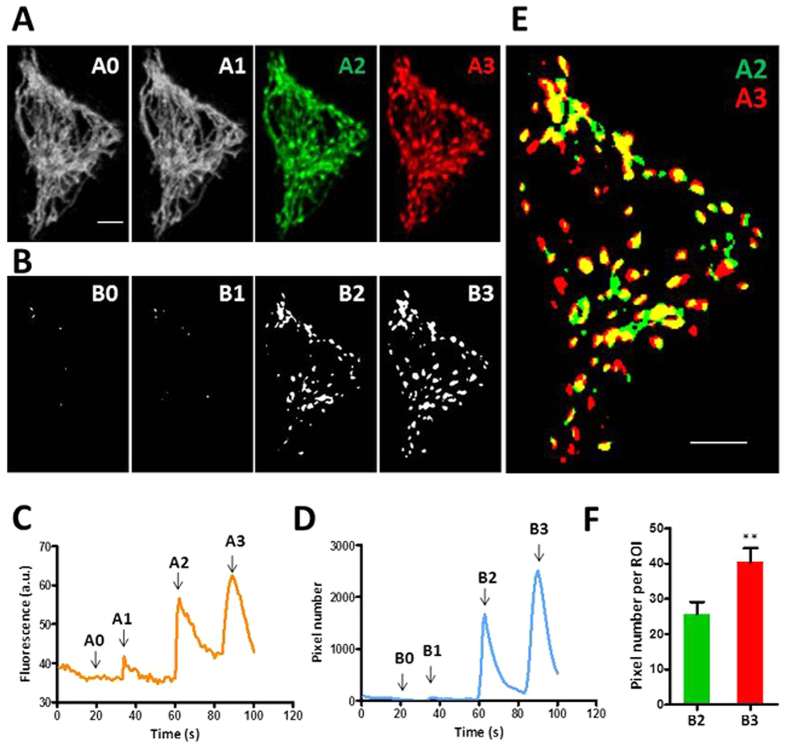
Local reversible swelling during whole-cell mitoflashes in Drp1-depleted cells. **(A)** A whole-cell mitochondrial reticulum viewed during quiescence (A0) and at peaks of three consecutive mitoflashes of growing intensity (A1–A3). Note that discrete local swelling sites were conspicuous in the last two mitoflash events, but not discernible in the first one. Scale bar, 5 μm. **(B)** Recruitment of swelling sites. A baseline image obtained during quiescence was subtracted from images in **A2** and **A3** and binary maps of swelling sites were generated by thresholding. **(C,D)** Time courses of mt-cpYFP and local swelling signals. Swelling signal is indexed by non-zero pixels in binary maps from time-lapse images using the algorithm in (**B**). **(E)** Local swelling sites in consecutive mitoflashes. Note that the vast majority of swelling sites seen in the second event (A2, color in green) were retained and grew bigger, while new swelling sites were also recruited in the third mitoflash (A3, color in red). **(F)** Average sizes of local swelling sites. n = 38–41 sites/group. **p < 0.01.

Another interesting observation is that mitochondrial reticulum exhibited reversible local swellings during a mitoflash (Fig. 6A and Supplementary information, Movie S2), similar to those previously reported by us^24^ and by others (termed “mitochondrial contraction”)^38^. These local swellings have been thought to be triggered by local transient openings of mPTP and thus provide an opportunity to characterize kinetics of mPTP gating in intact cells^24^. To this end, Fig. 6 shows a sequence of repetitive mitoflashes of progressively increasing amplitude in a whole-cell mitochondrial reticulum in a Drp1-KD cell. Local swelling sites were conspicuous at the second event and became more vigorous in the third event. Nearly all swelling sites in the second event re-appeared, while new sites were recruited in the third event (Fig. 6B,E). The nearest intervals between swelling sites range from 0.68 to 3.00 μm. Similar results were obtained in cells expressing the Drp1^K38A^ mutant (data not shown). These data indicate that mitoflash-associated local swelling is gradable and is associated with preferred locations of the reticular mitochondria. This result is consistent with the idea that transient mPTP opening plays an important role in the biogenesis of mitoflashes.

## Discussion

In this study, we have systematically examined possible roles of mitochondrial network dynamics in the regulation of mitoflash biogenesis and signaling. Based on data from a range of cell types with genetic manipulation of multiple dynamic proteins, including Mfn1, Mfn2, Opa1, Drp1 and Kif5b, our overall conclusion is that the rate of mitoflash occurrence in a given cell is independent of network reorganization by fission, fusion and motility, when metabolic contributions as well as detection bias are appraised and separated. In contrast, the magnitude of mitoflash signaling (signal mass) in a cell is dynamically regulated by network fragmentation and hyperfusion. These findings provides compelling evidence for the stochastic trigger model of mitoflash biogenesis^39^ and reveals a novel role for network dynamics in the regulation of mitochondrial signaling (see below).

### Constancy of mitoflash biogenesis in changing mitochondrial network

Our conclusion is based on critical synthesis of multiple lines of evidence. Experimental results from Mfn1, Mfn2 and Kif5b are straightforward: dramatic fragmentation or redistribution of mitochondrial network causes no detectable change in mitoflash frequency while exerting mild or no effect on mitochondrial respiration. However, cases with Opa1 and Drp1 appear to be counterintuitive and merit further analysis. In the absence of Opa1, we observed that spontaneous mitoflash activity was decimated, but not completely abolished, in fragmented network, dismissing Opa1 as an obligatory factor for mitoflash biogenesis^31^. However, this effect is unlikely attributable to altered mitochondrial morphology because similar network fragmentation in mitofusin-knockout cells caused no significant changes in mitoflash frequency. Instead, parallel experiments revealed that Opa1 deficiency markedly impairs mitochondrial respiratory function, in agreement with previous reports^32^,^33^. This superimposed metabolic change could have accounted for the lowered mitoflash activity because of the coupling of mitoflash biogenesis to metabolism^18^,^19^,^22^,^23^. To directly test this possibility, we verified that wild-type and *Opa1*^−/−^ MEFs exhibited similar mitoflash response to hyperosmotic stress. This result supports that spontaneous mitoflash activity in *Opa1*^−/−^ MEFs is altered by depressed respiration rather than network fragmentation.

In the situation with Drp1, there emerged a new population of events (miniflashes) that even outnumbered regular mitoflashes, such that the combined event frequency was much higher in Drp1-KD or Drp1^k38A^ cells. The emergence of miniflashes in hyperfused mitochondrial network was independently verified with different experimental approaches involving activation of mitofusin by S-3 and depletion of the fission factor Mff. Does this mean that hyperfused mitochondria generate new types of events? The answer is no. First, similar to regular mitoflashes, miniflashes were synchronous over fused mitochondrial network, and were also associated with transient loss of ΔΨ_m_. Second, miniflashes were sensitive to inhibition by ROS scavengers, mPTP inhibitor, and respiratory complex inhibitor, as regular mitoflashes did. Third, the amplitude and duration histograms of all events were fitted to single-exponential decaying functions, revealing no subpopulation of events. It is also noteworthy that these miniflashes closely resemble “precursor events” occurring at the rising feet of regular mitoflashes in cardiomyocytes^39^. Thus, we conclude that both miniflashes and regular mitoflashes are manifestations of the same stochastic process.

Then, do the Drp1-deficient cells exhibit hyperactive mitoflashes? The answer is unlikely when biases in detectability are taken into account. We showed that the noise level, quantified as SNR of basal mt-cpYFP fluorescence, was 5.2-fold higher in a whole-cell network (on average, 166 μm^2^ per mitoflash) than in a regular mitochondrial network (7.6 μm^2^ per mitoflash), as expected for the signal averaging effect. All else equal, greater SNR confers greater ability to identify small and brief events amidst noise. A fair and direct comparison should be limited to those events that are of larger amplitudes and duration and thus relatively insensitive to changes in detectability. In this regard, we found that the frequency of regular mitoflashes was unchanged in cells with hyperfused mitochondrial reticulum. Together with the constancy of respiration in these cells, this finding provides additional evidence supporting the conclusion that mitochondrial hyperfusion, while enhancing detectability and increasing observable mitoflash frequency, does not affect mitoflash biogenesis *per se*.

### Stochastic trigger model for mitoflash biogenesis

**Figure 7:**
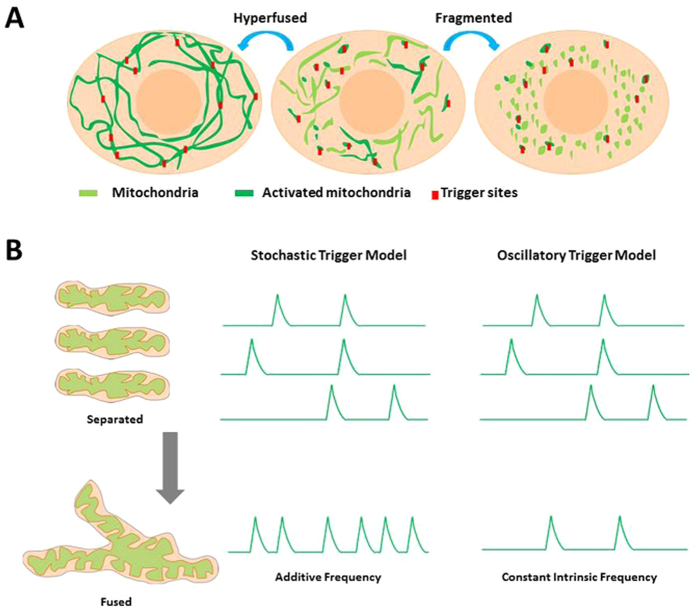
Model for regulating mitoflash biogenesis and signaling with reorganization of mitochondrial network. **(A)** Putative mitoflash trigger sites are marked with red blocks. Note that mitoflash productivity remains constant, while mitoflash area and hence total mitoflash signal mass change when the mitochondrial network hyperfuses or fragmentizes. **(B)** Stochastic trigger model versus oscillatory trigger model for mitoflash biogenesis. In the stochastic trigger model, hyperfused mitochondrial reticulum exhibits additive rate of mitoflash production. In contrast, oscillatory trigger model predicts synchronous oscillations at invariant frequency when the size of mitochondrial reticulum varies.

In a previous study we have proposed that, at the single-mitochondrion level, stochastic excitation of discrete trigger site (e.g., mPTP) signifies the ignition of mitoflash^39^. The constancy of mitoflash frequency at the single-cell level with altered mitochondrial dynamics leads us to further extend this model for the biogenesis of mitoflashes (Fig. 7A). Specifically, we propose that each cell contains a certain number of discrete trigger sites (N) that are randomly distributed throughout mitochondrial network. Different mitochondria or reticular networks operate independently and stochastically. When all intracellular mitochondria are considered as a whole, cell-level mitoflash frequency is proportional to the product NP_o_, where P_o_ is the probability of excitation of a trigger site. Whether network hyperfuses or fragmentizes, cell-level mitoflash frequency will be constant as long as N and respiration, which is an important determinant of P_o_, remain unchanged.

From the viewpoint of a single mitochondrion or a fused network, however, its number of trigger sites (n) increases additively with fusion, and hence its propensity of mitoflash production (nP_o_) is size-dependent. That is, a hyperfused mitochondrial reticulum should exhibit higher rate of mitoflash ignition; conversely, smaller mitochondria in pro-fission conditions should display reduced mitoflash frequency per mitochondrion. Indeed, both predictions were verified with our data from hyperfused or fragmented networks.

It should be noted that our results argue against an alternative model in which mitoflashes arise from intrinsic oscillation of the mitochondria^40^,^41^. This model predicts an invariant oscillation frequency when mitochondria hyperfuse or fragmentize (Fig. 7B). An exemplary intrinsic oscillator system can be found with cardiac pacemaking. An intact sinoatrial node consists of hundreds of pacemaker cells that are electrically coupled into a functional syncytium. The rate of action potentials of the whole system is similar to that of its constituent pacemaker cells in isolation^42^, in sharp contrast to the additive behavior of mitoflash biogenesis when mitochondria hyperfuse or fragmentize.

The hypothesis on the existence of discrete trigger sites is also in agreement with possible role of mPTP in mitoflash ignition^16^,^17^. In this study, data shown in Fig. 6 provide vivid details that match with known characteristics of mPTP: multiple states of increasing conductance of mPTP may be progressively recruited during sustained and repetitive activities^43^. Given that open probability of mPTP is modulated by diverse endogenous and exogenous factors, this stochastic trigger model also provides a unifying framework for understanding the FM regulation of mitoflashes.

### Regulation of mitoflash signaling by network dynamics

While previous studies have established various FM mechanisms of mitoflash regulation^17^, the present study represents the first report of a primarily magnitude-modulatory mechanism whereby dynamic reorganization of mitochondrial network regulates the total mass of mitoflash signaling. Specifically, mitoflash frequency is largely independent of network dynamics; however, the average area of individual mitoflashes, and hence the mitoflash signal mass, is directly controlled by network fragmentation and hyperfusion. That is, mitochondrial dynamics constitute a magnitude-modulatory mechanism to regulate the total mass of mitoflash signals, independently of mitoflash frequency. That mitoflash frequency remains constant when network reorganizes indicates that this magnitude-modulatory mechanism is orthogonal to FM mechanisms, such that mitoflash signaling can be dynamically and independently regulated in both FM and magnitude-modulatory modes. Taken together, these findings provide new insights into roles of mitochondrial dynamics in the regulation of mitochondrial function and signaling.

## Materials and Methods

### Reagents

Small molecule S-3 was gifted by Professor Quan Chen^37^. D-glucose, sorbitol, menadione, mitoTEMPO, cyclosporine A (CsA), and rotenone were from Sigma. Tetra-methylrhodamine methyl ester (TMRM) was from Invitrogen. SS31 (D-Arg-Dmt-Lys-Phe-NH2) was synthesized as described^44^. Antibody against Drp1 was obtained from BD Biosciences.

### Plasmids and adenoviral vectors

The dominant-negative Drp1^K38A^ mutant was inserted into pcDNA4-TO vector (Invitrogen). DNA fragment of mt-cpYFP or mitoSypHer was inserted into pENTR/TEV/D-TOPO vector (Invitrogen) for adenovirus and the adenoviral expression system (Invitrogen) was used for infection.

### Cell culture

NRK cell line was gifted by Professor Li Yu^15^. Stable expression of mt-cpYFP in HeLa cells was established as previously described^24^. MEFs, NRK cells, and HeLa cells were all maintained in Dulbecco Minimal Essential Medium (DMEM) containing 10% fetal bovine serum (Hyclone) and 1% penicillin and streptomycin (Invitrogen) and incubated at 37 °C in a humidified atmosphere of 5% CO_2_.

### Gene silencing, transfection and infection

RNAi sequences (sense: GGCUAGCCAGAGAAUUACCdTdT, antisense: GGUAAU UCUCUGGCUAGCCdTdT for human *Drp1*; sense: CGCUGACCUGGAACAAGG AdTdT, antisense: UCCUUGUUCCAGGUCAGCGdTdT for human *Mff* ) were used. All siRNAs were custom-synthesized products. The negative control was used as control for all siRNA experiments. The double-stranded siRNA was dissolved in DEPC-treated water. Cells were incubated with 100 nM siRNA or 2 μg plasmid in 2 mL of OPTI-MEM Reduced Serum Medium (Life Technologies) containing Lipofectamine RNAiMax (Invitrogen) or Lipofectamine 2000 (Invitrogen), respectively. For mitoflash detection, cells were infected with adenovirus carrying mt-cpYFP at a multiplicity of infection (MOI) of 20 and experiments were performed after 48–72 h culture.

### Confocal microscopy and image processing

Confocal imaging was carried out with a Zeiss LSM710 microscope with a 40×, 1.4 NA oil-immersion objective. Time-lapse imaging of mt-cpYFP fluorescence was performed by excitation at 488 and 405 nm, and emission collection at >505 nm. MitoSypHer was excited at 405 and 488 nm with emission collection at >505 nm. When cells were simultaneously loaded with TMRM (Invitrogen) at 37 °C for 10 min, multitrack scanning was performed with excitation at 488 and 405 nm for mt-cpYFP and 543 nm for TMRM, and fluorescence emissions was collected at 505–530, 505–530 and >560 nm, respectively. Usually 100 frames were acquired at 1 s/frame, and the axial resolution was set to 1.0 μm. In some experiments, linescan imaging was performed at 5 ms/line (Fig. 4C) or 763 μs/line (Supplementary information, Figure S6). The mt-cpYFP images were also used for visualization of mitochondrial network and subcellular localization. When needed, cell boundary was determined by differential interference contrast (DIC) images. All experiments were carried out with Tyrode’s solution (137 mM NaCl, 5.4 mM KCl, 1.2 mM MgCl_2_, 1.2 mM NaH_2_PO_4_, 1.8 mM CaCl_2_, 10 mM glucose, and 20 mM HEPES, pH 7.35, adjusted with NaOH) at room temperature (22–26 °C). Confocal images were analyzed using Interactive Data Language (IDL, Research Systems) software and customer-devised programs.

### Mitoflash Terminology

For morphological classification, we sorted mitoflashes into punctiform (round and oval-shaped), tubular (tubule-shaped) and reticular (tubular with branches) subgroups. In Drp1-deficient cells, we refer mitoflash in mitochondria that hyperfused into a single functional unit as “whole-cell reticulum” or “whole-cell network”. For parametric quantification, we measured the amplitude (ΔF/F_0_, dimensionless), spatial area (A, μm^2^), full-duration at half maximum (FDHM, s), and signal mass (ΔF/F_0_ × A × FDHM, μm^2^·s) for individual events. Those with ΔF/F_0_ < 0.15 and FDHM < 4 s are also called “miniflashes”.

### Measurement of mitochondrial respiratory function in intact cells

Cells were cultured in XF24 cell-culture microplates (Seahorse Bioscience) at 4.5–5 × 10^4^ cells/well in DMEM supplemented with 10% FBS for 24 h. Then the culture medium was changed to assay medium supplemented with 25 mM glucose and 2.5 mM pyruvate (pH 7.4) and incubated for 1 h at 37 °C prior to measurement. Bioenergetic analyses were performed in an XF24 Extracellular Flux Analyzer (Seahorse Bioscience) with oligomycin (1 μM), FCCP (1 μM), rotenone (1 μM), and antimycin A (AA, 1 μM) injected sequentially. Cellular oxygen consumption rate (OCR) was normalized relative to the number of cells in each well.

### Immunoblotting

Cells were lysed in lysis buffer (30 mM HEPES, 100 mM NaCl, 0.5% Nonidet P (NP)-40, protease inhibitors mixture, pH 7.6) on ice for 10 min and the lysates were centrifuged at 13,000 rpm for 10 min. Proteins were resolved by SDS-PAGE transferred onto nitrocellulose membranes and detected with the indicated antibodies. Blots were visualized using secondary antibodies conjugated with IRDye (LI-COR) by an Odyssey imaging system (LI-COR).

### Statistical analysis

The data are expressed as mean ± SEM, and Student’s t test was applied to determine statistical significance and p < 0.05 was considered statistically significant.

## Additional Information

**How to cite this article**: Li, W. *et al*. Regulation of Mitoflash Biogenesis and Signaling by Mitochondrial Dynamics. *Sci. Rep.* **6**, 32933; doi: 10.1038/srep32933 (2016).

## Supplementary Material

Supplementary Information

Supplementary Movie S1

Supplementary Movie S2
