# Supplementary material for: Regulation of Mitoflash Biogenesis and Signaling by Mitochondrial Dynamics
Source: Sci Rep. 2016 Sep 13;6:32933. doi: 10.1038/srep32933 (PMC5020656; doi:10.1038/srep32933)
Supplement: Supplementary Information [file srep32933-s1.pdf]

Supplementary Information

Regulation of Mitoflash Biogenesis and Signaling by Mitochondrial Dynamics

Wenwen Li[1], Tao Sun[1], Beibei Liu[1], Di Wu[1], Wenfeng Qi[1], Xianhua Wang[1], Qi Ma[1,2], Heping Cheng[1]

[1]State Key Laboratory of Membrane Biology, Beijing Key Laboratory of Cardiometabolic Molecular Medicine, Peking-Tsinghua Center for Life Sciences, Institute of Molecular Medicine, Peking University, Beijing, China.

[2]To whom correspondence should be addressed. E-mail: maq@pku.edu.cn

Figure S1. **(A-C)** Mitoflash amplitude (A), duration (B) and area (C) in wild-type (WT), *Mfn1*$^{-/-}$, *Mfn2*$^{-/-}$, and Mfn1/2 DKO MEFs. n = 25-31 mitoflashes per group. ***, p < 0.001 vs WT cells. **(D)** Histograms of mitoflash amplitude ($\Delta F/F_0$) in WT and Mfn1/2 DKO MEFs.

Figure S2. **(A)** Examples of punctiform, tubular and reticular mitoflashes in wild-type (WT) NRK cells. Image horizontal size, 2.5 μm. **(B)** Average sizes for the three subgroups of mitoflashes in WT and Kif5b-knockout NRK cells. n = 17-25 mitoflashes per group.

Figure S3. **(A-D)** Effects of Opa1 on mitoflash amplitude (A), duration (B), area (C) and signal mass (D). n = 25-27 mitoflashes per group. ***, p < 0.001. **(E)** Averaged time courses of spontaneous and hyperosmosis-stimulated mitoflashes in *Opa1*$^{-/-}$ MEFs. n = 11-25 mitoflashes per group. Experimental conditions were the same as in Figure 3C.

Figure S4. Mitoflashes detected by pH indicator mitoSypHer in Opa1-knockout (KO) cells. **(A)** Mitochondrial morphology in Opa1-KO cells expressing mitoSypHer. Scale bar, 5 μm. **(B)** Time courses of mitoSypHer-reported mitoflash. Note that mitoSypHer fluorescent transients at 488 and 405 nm excitation were accompanied by a TMRM-reported mitochondrial depolarization. Insets show enlarged time-lapse images of the mitoflash with horizontal size of 2.5 μm. **(C)** Effect of Opa1 deficiency on spontaneous and hyperosmosis-stimulated mitoflash activity reported by mitoSypHer. n = 33-38 cells per group. ***, p < 0.001.

Figure S5. **(A)** Mitoflash frequencies were inhibited by respiratory chain inhibitors. N = 20-30 cells per group. **(B)** Effect of the inhibitors on basal OCR. n = 3 independent experiments. **(C)** Low mitochondrial membrane potential of *Opa1*-null cells. n = 8-9 dishes of cells per group. **(D)** Effect of the inhibitors on membrane potential. n = 5-15 dishes of cells per group. *, p < 0.05; **, p < 0.01; ***, p < 0.001.

Figure S6. Synchronous ignition of mitoflashes across whole-cell mitochondrial reticulum, as resolved by fast linescan imaging in Drp1-KD HeLa cells. **(A)** Placement of the scanning line. **(B)** Linescan image obtained at the speed of 763 μs/line. **(C)** Time courses of mitoflash ignition at four loci separated up to 18 μm apart.

Figure S7. Miniflashes in hyperfused mitochondria. **(A)** Representative shapes of mitochondria and time courses of mitoflashes in S-3-treated or Mff-knockdown (KD) HeLa cells. Upper panels: confocal images of mitoflashes in whole-cell mitochondrial reticulum at their peak intensities. Scale bar, 5 μm. Bottom panels: corresponding time course of simultaneously recorded mt-cpYFP and TMRM signals. **(B)** Mitoflash frequency in wild-type (WT) and Mff-KD cells. n = 58-76 cells in each group. ***, $p < 0.001$. **(C)** Event frequency in WT and Mff-KD cells. n = 29-43 events in each group. NS, no significance; ***, $p < 0.001$.

Movie S1. Mitoflashes and miniflashes in Drp1-knockdown HeLa cells.

Movie S2. Local mitochondrial swellings during mitoflashes in a Drp1-knockdown HeLa cell.

Table S1. An estimate of electrical length constant of tubular mitochondria.

Parameter	Description	Value	Unit
d_{mito}	Diameter of mitochondrion	0.5 [1]	μm
ρ_{mt}	Mitochondrial internal resistivity	10 §	$\Omega \cdot m$
g_{mem}	Mitochondrial membrane conductivity	0.63662*	$\Omega^{-1} \cdot m^{-2}$
ε	Cristae folding factor	3	Dimensionless
λ	Electrical length constant	82#	μm

§ $\sigma_{1i} = (1/\rho_{mt}) \in [0.1,1][S/m]$ [2], choose the maximal value of $\rho_{mt} = 1/0.1 = 10\Omega \cdot m$

*$g_{mem} = \sigma_{1m}/(\pi d_{mito})$, $\sigma_{1m} \in [10^{-8}, 10^{-5}][S/m]$ [2], choose an intermediate value of $g_{mem} = 10^{-6}/\pi d_{mito}$

cable theory [3] , $\lambda = \sqrt{d/(4g\rho)}$, apply to mitochondrion case, $\lambda = \sqrt{\frac{d_{mito}}{4\varepsilon g_{mem}\rho_{mt}}}$.

Reference

1. Alberts, B., *et al. Molecular biology of the cell*, (Garland Science, New York, 2002).
2. Kotnik, T. & Miklavcic, D. Theoretical evaluation of voltage inducement on internal membranes of biological cells exposed to electric fields. *Biophysical journal* **90**, 480-491 (2006).
3. Brzychczy, S. & Poznanski, R.R. Chapter 12 - Reaction-Diffusion Equations. in *Mathematical Neuroscience* 151-163 (Academic Press, Boston, 2014).

S1

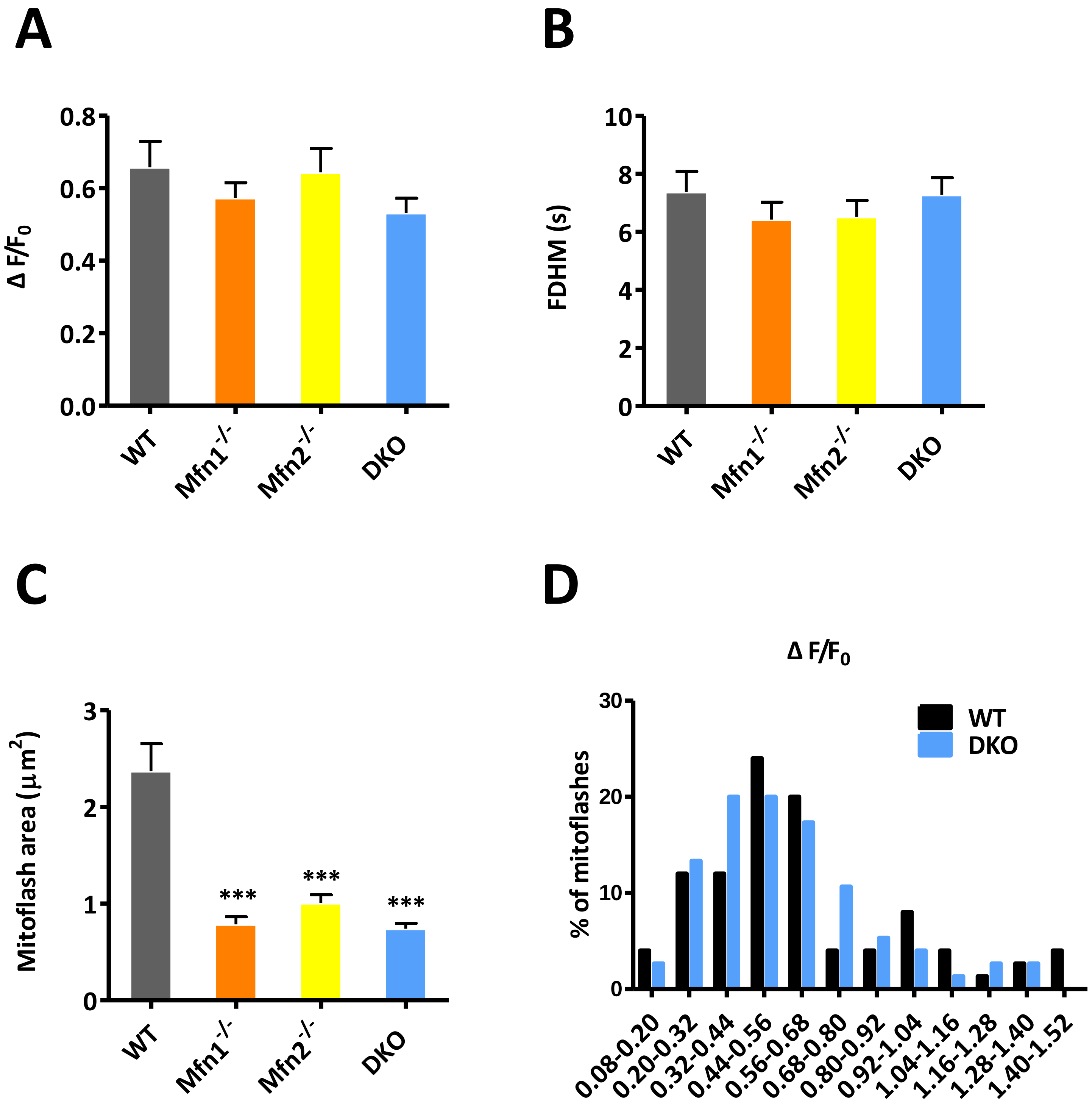

A
0.8
0.6
0.4
0.2
0.0
Δ F/F0
WT
Mfn1-/-
Mfn2-/-
DKO
B
10
8
6
4
2
0
FDHM (s)
WT
Mfn1-/-
Mfn2-/-
DKO
C
3
2
1
0
Mitoflash area (μm2)
***
***
***
WT
Mfn1-/-
Mfn2-/-
DKO
D
Δ F/F0
30
20
10
0
% of mitoflashes
WT
DKO
0.08-0.20
0.20-0.32
0.32-0.44
0.44-0.56
0.56-0.68
0.68-0.80
0.80-0.92
0.92-1.04
1.04-1.16
1.16-1.28
1.28-1.40
1.40-1.52

S2

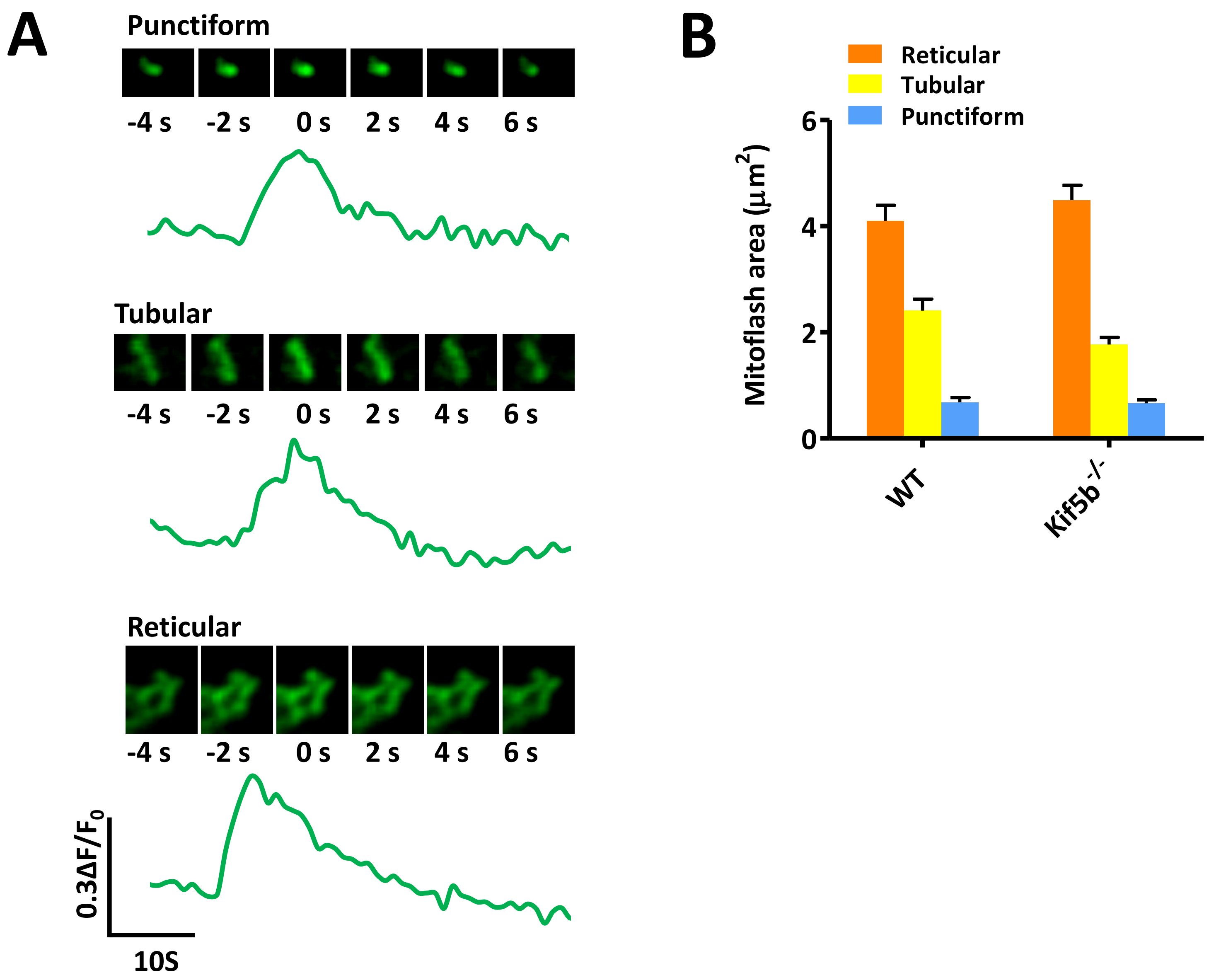
A
Punctiform
-4 s
-2 s
0 s
2 s
4 s
6 s
Tubular
-4 s
-2 s
0 s
2 s
4 s
6 s
Reticular
-4 s
-2 s
0 s
2 s
4 s
6 s
0.3ΔF/F0
10S
B
Reticular
Tubular
Punctiform
Mitoflash area (μm2)
6
4
2
0
WT
Kif5b -/-

S3

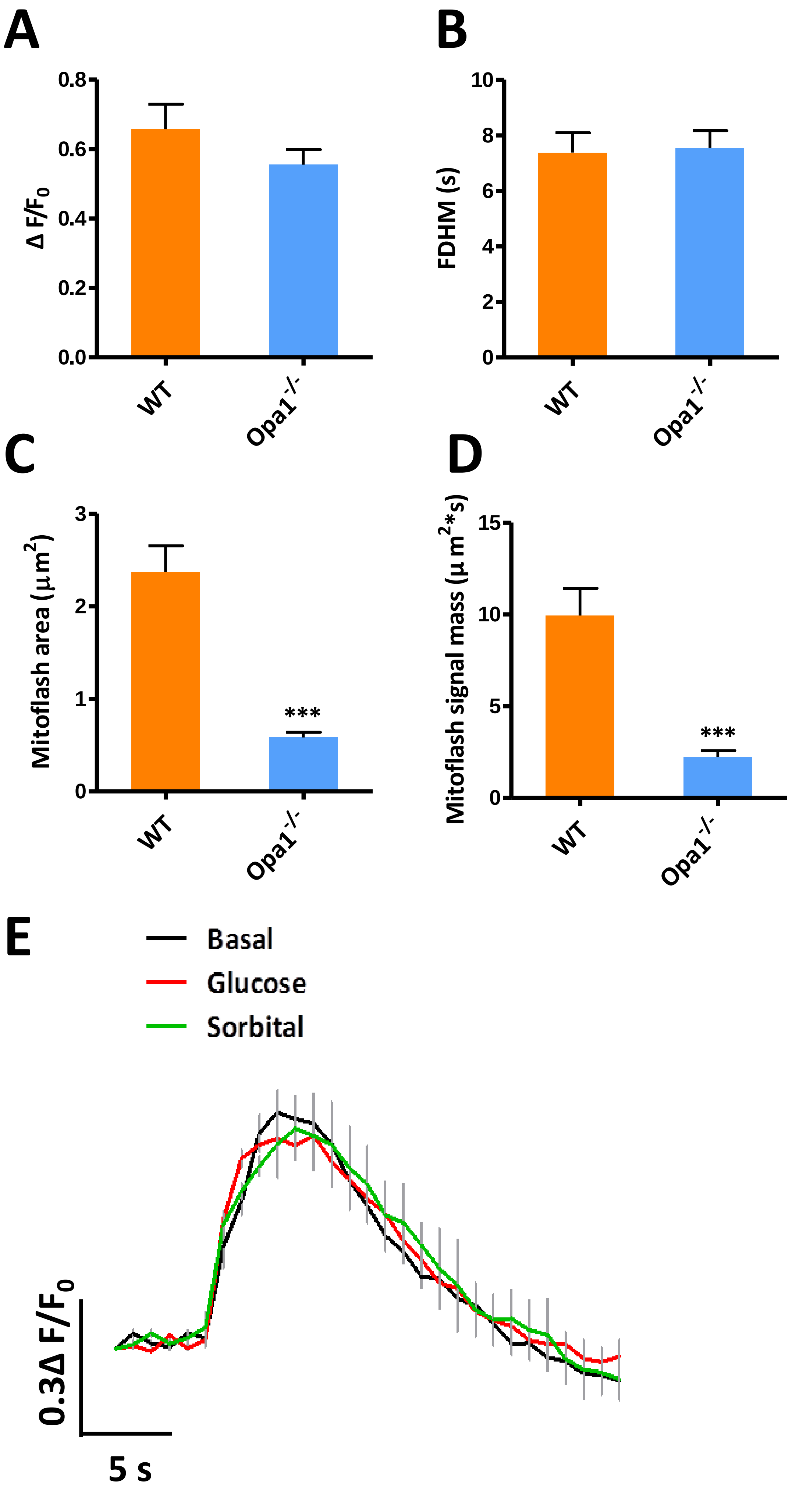

A
0.8
0.6
0.4
0.2
0.0
Δ F/F0
WT
Opa1 -/-
B
10
8
6
4
2
0
FDHM (s)
WT
Opa1 -/-
C
3
2
1
0
Mitoflash area (μm2)
***
WT
Opa1 -/-
D
15
10
5
0
Mitoflash signal mass (μm2*s)
***
WT
Opa1 -/-
E
Basal
Glucose
Sorbital
0.3Δ F/F0
5 s

S4

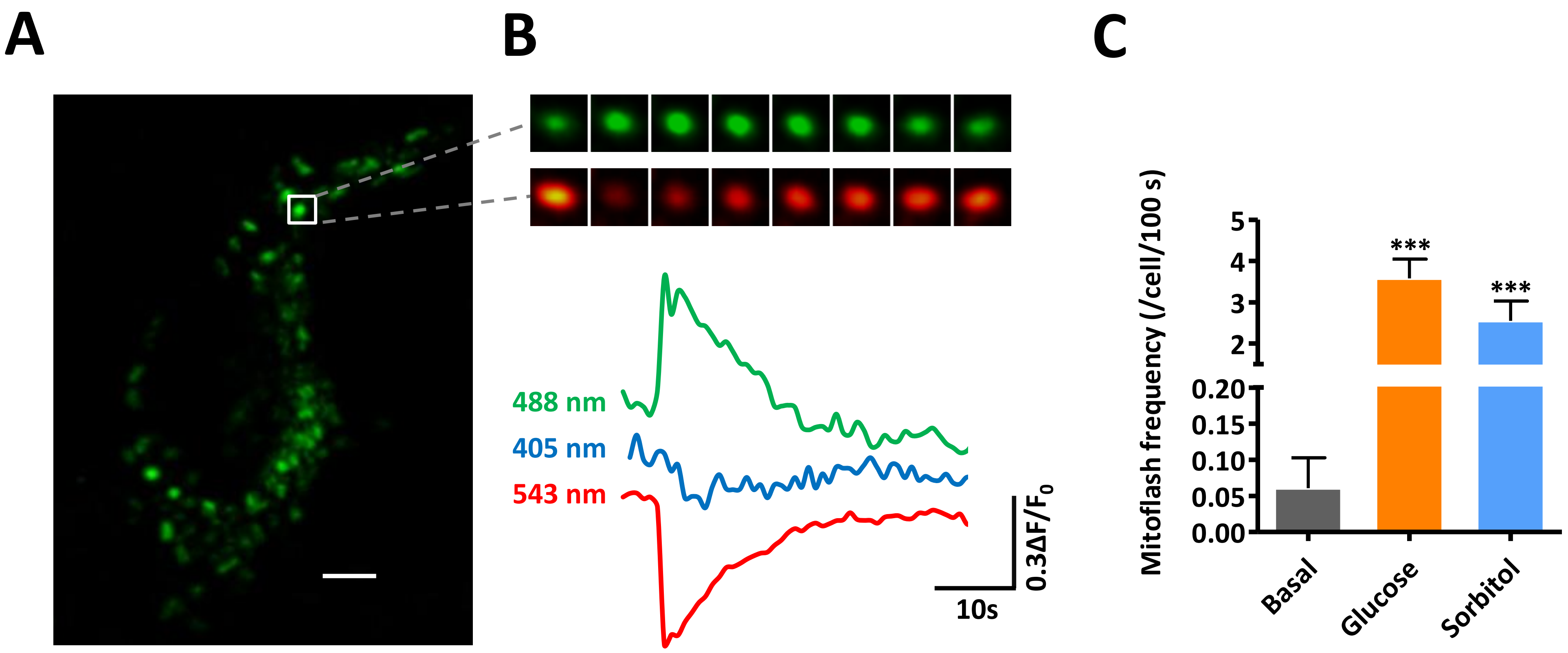

A
B
C
488 nm
405 nm
543 nm
0.3ΔF/F0
10s
Mitoflash frequency (/cell/100 s)
5
4
3
2
0.20
0.15
0.10
0.05
0.00
***
***
Basal
Glucose
Sorbitol

S5

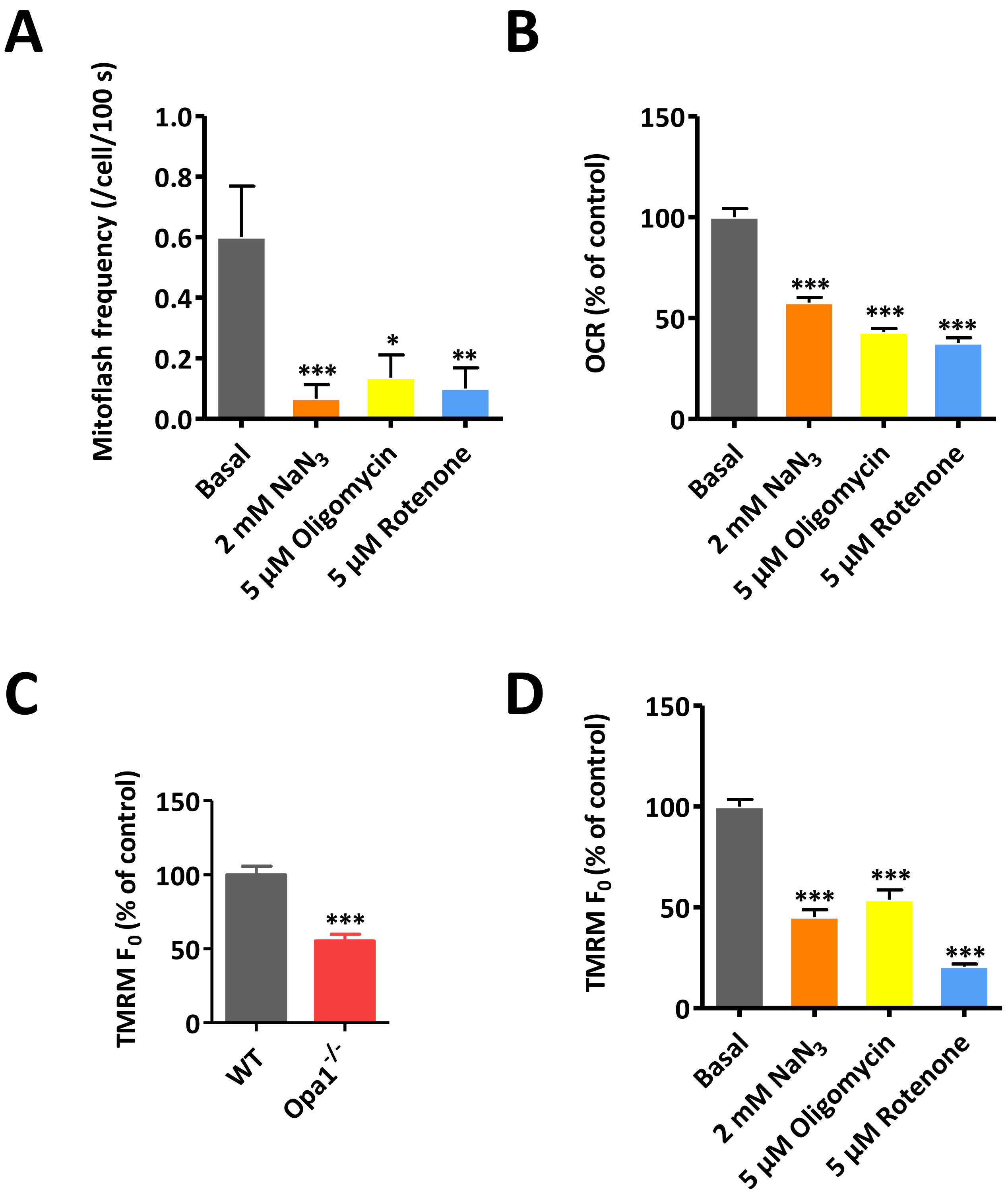

A
Mitoflash frequency (/cell/100 s)
1.0
0.8
0.6
0.4
0.2
0.0
***
*
**
Basal
2 mM NaN3
5 µM Oligomycin
5 µM Rotenone
B
OCR (% of control)
150
100
50
0
***
***
***
Basal
2 mM NaN3
5 µM Oligomycin
5 µM Rotenone
C
TMRM F0 (% of control)
150
100
50
0
***
WT
Opa1-/-
D
TMRM F0 (% of control)
150
100
50
0
***
***
***
Basal
2 mM NaN3
5 µM Oligomycin
5 µM Rotenone

S6

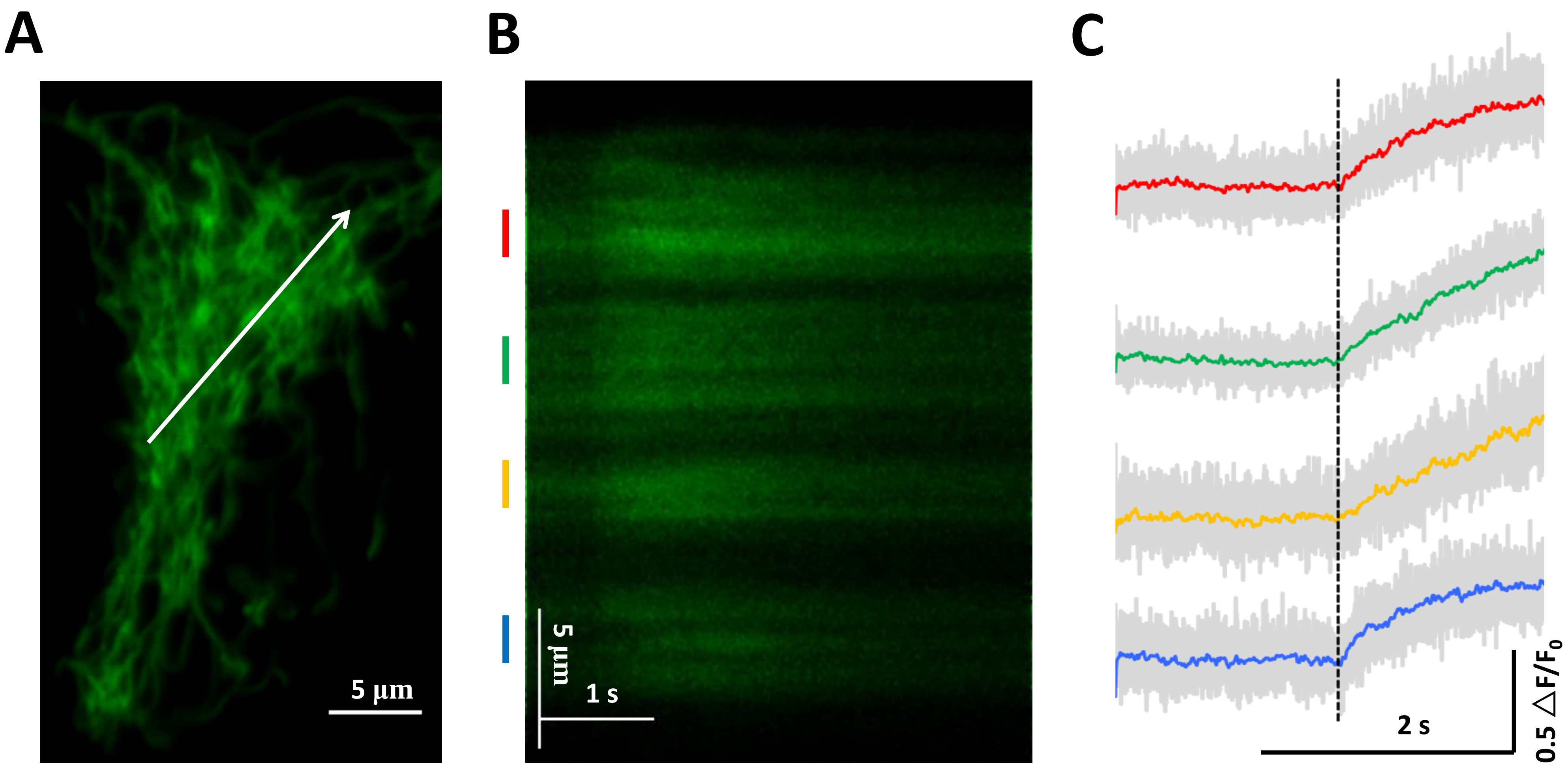

S7

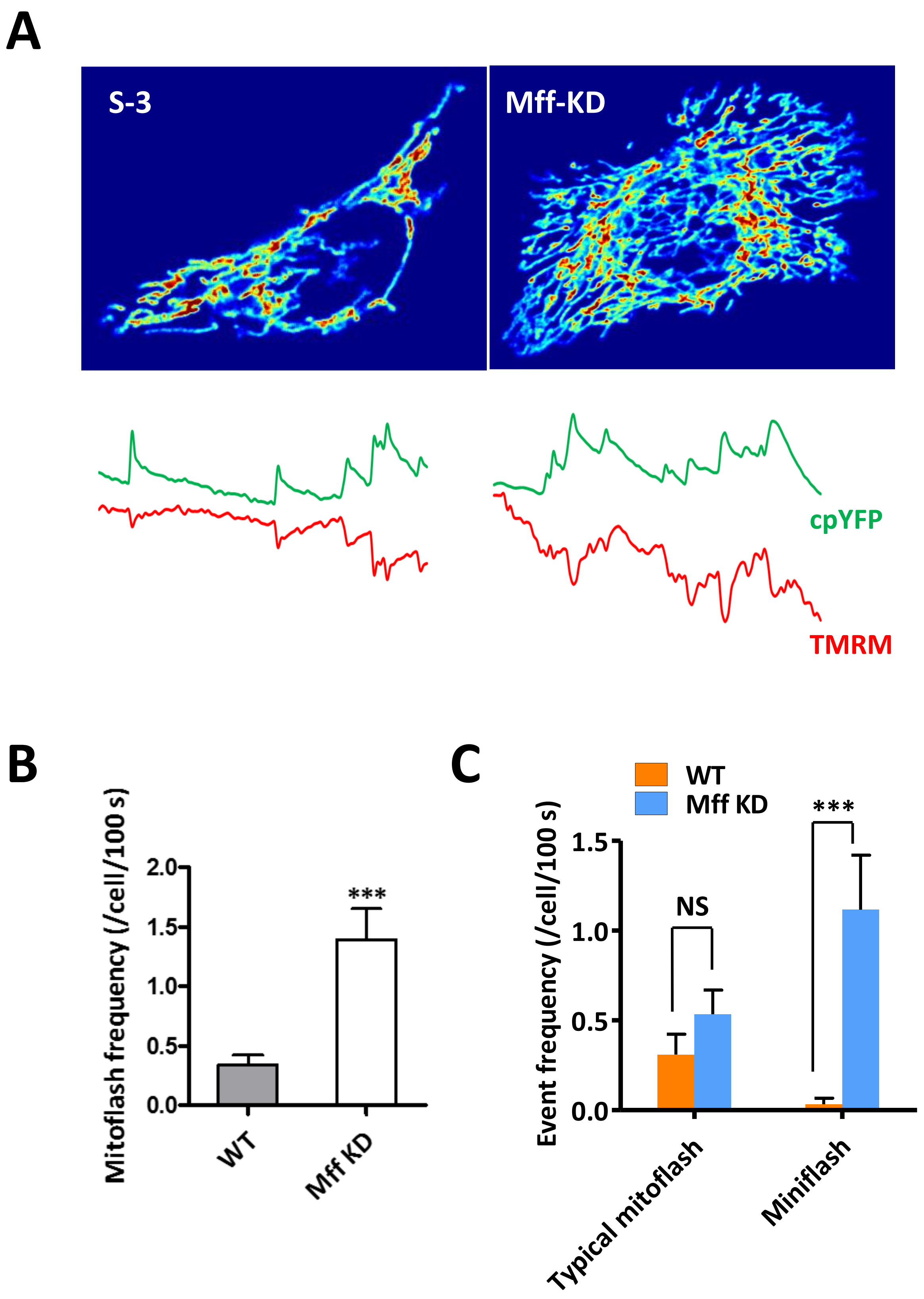
A
S-3
Mff-KD
cpYFP
TMRM
B
Mitoflash frequency (/cell/100 s)
2.0
1.5
1.0
0.5
0.0
***
WT
Mff KD
C
WT
Mff KD
Event frequency (/cell/100 s)
1.5
1.0
0.5
0.0
NS
***
Typical mitoflash
Miniflash
